# Contribution of the neighborhood environment to cross-sectional variation in long-term CVD risk scores in the Framingham Heart Study

**DOI:** 10.1371/journal.pone.0201712

**Published:** 2018-08-06

**Authors:** Todd R. Sponholtz, Ramachandran S. Vasan

**Affiliations:** 1 Section of Preventive Medicine and Epidemiology, Boston University School of Medicine, Boston, MA, United States of America; 2 National Heart, Lung, and Blood Institute, Bethesda, MD, United States of America; 3 Framingham Heart Study of the National Heart, Lung, and Blood Institute and Boston University School of Medicine, Framingham, MA, United States of America; Medizinische Universitat Innsbruck, AUSTRIA

## Abstract

Few studies of the health impact of the built environment have examined downstream outcomes, such as cardiovascular disease. We analyzed the neighborhood-level proportional variance in the 10- and 30-year Framingham risk scores (FRS) in the Framingham Heart Study. Our analysis included 3,103 Offspring- and Generation 3 cohort participants 20–74 years old, inhabiting private residences in Massachusetts geocoded to neighborhoods (defined as 2000 US Census block groups) containing the residences of ≥5 participants. The outcome variables were log-transformed to mitigate the effects of the non-normal distributions. In order to remove the possible effects of neighborhood clustering by age and sex, we analyzed residuals of the transformed FRS regressed upon age and sex. Neighborhood-level intraclass correlations (ICCs) and 95% confidence intervals (CIs) of age- and sex-independent, log-transformed FRS were estimated using multilevel linear regression. Individual- and neighborhood-level variables were then added to models to evaluate their influence on ICCs. Analyses were repeated stratified by sex. Among 2,888 participants living in 187 neighborhoods, 1.73% (95% CI: 0.62, 4.72%) of the variance in 10-year FRS was explained at the neighborhood level. The neighborhood ICC was 2.70% (95% CI: 0.93, 7.56) among women but 0.23% (95% CI: 0.00, 99.47%) among men. In the analysis of the neighborhood-level variances in 30-year FRS among 2,317 participants residing in 164 neighborhoods, the ICCs were 3.31% (95% CI: 1.66, 6.47%), 6.47% (95% CI: 3.22, 12.58), and 0.74% (95% CI: 0.01, 33.31), among all participants, women, and men, respectively. In our homogenous middle-class white population in Massachusetts, residential neighborhoods explained a small proportion of the variance in CVD risk.

## Introduction

The built environment has generated a great deal of interest as a potential causative factor the epidemic of obesity, and in the incidence of cardiovascular disease. Recent studies have reported associations between aspects of the built environment and outcomes such as diet, physical activity, obesity, and diabetes [1, 2]. However, relatively few studies have investigated the impact of the built environment on downstream outcomes like cardiovascular disease (CVD) [3–6]. These studies were all conducted in Europe and the amounts of variation in CVD risk attributable to neighborhoods reported have tended to be modest [3–5]. We estimated the proportional variance in the Framingham 10-year (short-term) [7] and 30-year (long-term) [8] CVD risk scores (FRS) attributable to US Census block group of residence among participants in the Framingham Heart Study, a community-based US cohort study.

## Materials and methods

The current analysis used data from the Framingham Heart Study Offspring- and Generation 3 (Gen 3) cohorts, which were initiated in 1971 and 2002, respectively. Details of recruitment and data collection for these cohorts have been described previously [9, 10]. Our analysis includes participants attending Offspring cohort examination 7 (1998–2001) and Gen 3 cohort examination 1 (2002–2005). All participants provided written informed consent and the Institutional Review Board of the Boston Medical Center approved the study protocol.

The home addresses of Offspring- and Generation 3 cohort members at the time of examination 7 (Offspring cohort, 1998–2001) or examination 1 (Gen 3 cohort, 2002–2005) were geocoded to 2000 US Census block groups (henceforth referred to as neighborhoods). Data on years of education were collected at examination 2 for Offspring cohort participants and examination 1 for Gen3 cohort members. At examination 7 (Offspring cohort) and examination 1 (Gen 3 cohort), participants completed a questionnaire on demographics, lifestyle, and medication history. Weight and height were measured at each examination according to a standardized protocol. Body mass index (BMI) was calculated as weight (kg)/height (m)^2^. Blood pressure was measured two times in the right arm, at least 5 minutes apart, using a mercury sphygmomanometer. The average of the two measurements was used in the analysis. A blood sample was obtained, and standardized assays were used to measure total serum cholesterol, high-density lipoprotein (HDL), and glucose. Diabetes was defined as fasting blood glucose ≥126 mg/dL or current use of anti-diabetic medication.

Ten-year- [7] and thirty-year [8] Framingham cardiovascular risk scores (FRS) were determined on the basis of sex, age, systolic blood pressure (SBP), anti-hypertensive medication use, smoking, diabetes, serum total cholesterol, and HDL. The FRS were log-transformed in to reduce the effects of non-normality. In order to remove the potential contributions of neighborhood clustering by age and sex on the neighborhood-level variance, the outcome variables used in the analysis were standardized residuals of the natural logarithm-transformed FRS regressed on age and sex.

There were 7,634 participants who attended the relevant examinations (3,539 from the Offspring cohort and 4,095 from the Gen 3 cohort). For the analysis of the 10-year FRS, we excluded 850 participants whose age was not in the validated range for the score (30–74 years). Additionally, we excluded 413 due to prevalent CVD, 37 who did not report living in a private residence, and 205 for whom data to calculate the 10-year FRS was not available. We limited analysis to Massachusetts neighborhoods which contained ≥5 participants who were eligible for inclusion. A further 3,241 participants were excluded for this reason. Thus, 2,888 participants inhabiting 187 neighborhoods were available for the analysis of this outcome. For the analysis of the 30-year FRS, we excluded 1,972 participants whose age was not between 20 and 59 years, 131 with prevalent CVD, 9 who did not report living in a private residence, 120 for whom data to calculate the 30-year FRS was not available, and 3,085 who did not live in a Massachusetts neighborhood with 5 or more eligible participants. Our analytic sample for this outcome was 2,317 participants living in 164 neighborhoods. The sex-stratified analyses included 1,053 men in 94 neighborhoods with ≥5 men who were eligible for inclusion and 1,374 women in 117 neighborhoods with ≥5 eligible women for 10-year FRS; the analysis of the 30-year FRS included 837 men in 84 neighborhoods and 1,074 women in 101 neighborhoods.

We estimated the cross-sectional intraclass correlation coefficients (ICCs), and their 95% confidence intervals (CIs) using multilevel linear regression models [11, 12]. Multilevel linear regression uses the differences between the values of the outcome among the individual observations, the means of each second level unit (in the present analysis, the neighborhoods) within which individual observations are grouped, and the overall sample mean of the outcome variable to apportion the total amount of variance in the sample among the individual and group levels. The ICC represents the proportion of the total variance attributable to the group level [12]. It thus can range from 0 to 1, with lower values indicating less of the variance in the outcome is attributable to the group level [12]. We first fit “empty” models, with no predictors, to determine the proportion of the total variation in FRS attributable to the neighborhood levels. Individual-level and neighborhood-level factors were then added, one at a time, to the model to observe their influence on the group-level FRS variance explained at the neighborhood level. All covariates were then included in a single model. If a portion of the neighborhood-level variation is due to clustering of individuals’ FRS-associated characteristics within neighborhoods, addition of these characteristics will account for some of the neighborhood-level variance, and the ICC will be reduced [13]. Similarly, the addition of neighborhood characteristics that vary within the sample of neighborhoods and influence the outcome to the model will also reduce the ICC [14].

Individual-level covariates included education (≤12, 13–15, 16, ≥17 years), body mass index (<18.5, 18.5–24, 25–29, 30–34,35–39, ≥40 kg/m^2^), waist circumference (quartiles), physical activity index (quartiles), [15] and depression (Center for Epidemiological Studies of Depression Scale [CES-D] score dichotomized at ≥16) [16, 17]. The neighborhood-level covariates were comprised of the percentage of green space, number of food stores, and number of intersections within the neighborhood (all modeled as quartiles). We repeated analyses stratified by sex.

All analyses were conducted using SAS software, version 9.4 (SAS Institute, Inc., Cary, North Carolina).

## Results

Among the 2,888 participants included in the main analysis of the 10-year FRS, the mean age was 50.6 years (Table 1). The proportions of overweight and obese participants were 39.0% and 28.7%, respectively, and the mean waist circumference was 38.2 inches. Over 16% had a CES-D score ≥16. Men had higher blood pressure and serum total cholesterol levels, a greater prevalence of blood pressure medication use and diabetes, and lower circulating HDL levels. They were also more highly educated, more physically active, more likely to work outside the home, and had a higher BMI and waist circumference. Characteristics of 2,317 participants included in the analysis of the 30-year FRS were younger (mean 43.9 years) and less likely to lack employment outside the home (13.1% vs. 24.1%), but were otherwise similar.

**Table 1:** Characteristics of framingham offspring and generation 3 participants at offspring cohort examination 7 (1998–2001)/generation 3 examination 1 (2002–2005).

	All participants		Women		Men	
	10 year FRS	30 year FRS	10 year FRS	30 year FRS	10 year FRS	30 year FRS
N	2888	2317	1374	1074	1053	837
Age, years	50.6 (11.4)	43.9 (9.6)	51.1 (11.5)	44.0 (9.8)	50.4 (11.4)	44.0 (9.5)
Male sex	1291 (44.7)	1042 (45.0)	0 (0.0)	0 (0.0)	1053 (100.0)	837 (100.0)
Systolic blood pressure, mm Hg	121.7 (17.0)	118.0 (14.7)	119.7 (17.6)	115.2 (14.9)	124.1 (16.1)	121.3 (13.9)
Current user of antihypertensive Medication, n (%)	563 (19.5)	274 (11.8)	263 (19.1)	124 (11.6)	210 (19.9)	104 (12.4)
Total cholesterol, mg/dL	196.3 (35.4)	193.3 (35.4)	197.5 (36.3)	191.7 (36.0)	195.0 (34.3)	195.2 (34.6)
High-density lipoprotein, mg/dL	53.3 (15.7)	53.4 (15.5)	59.3 (15.6)	59.6 (15.4)	46.3 (12.7)	46.5 (12.1)
Glucose, mg/dL	99.7 (23.3)	97.1 (21.7)	96.7 (22.7)	94.2 (22.4)	102.9 (23.1)	100.7 (21.8)
Diabetes, n (%)	209 (7.2)	102 (4.4)	84 (6.1)	35 (3.3)	83 (7.9)	47 (5.6)
Current smoker, n (%)	509 (17.6)	470 (20.3)	249 (18.1)	220 (20.5)	188 (17.9)	173 (20.7)
Education, years, n (%)						
≤12	925 (32.0)	589 (25.4)	459 (33.4)	263 (24.5)	335 (31.8)	226 (27.0)
13–15	680 (23.6)	556 (24.0)	338 (24.6)	275 (25.6)	231 (21.9)	186 (22.2)
16	608 (21.1)	467 (20.2)	272 (19.8)	220 (20.5)	236 (22.4)	173 (20.7)
≥17	570 (19.7)	646 (27.9)	254 (18.5)	287 (26.7)	218 (20.7)	232 (27.7)
Missing	105 (3.6)	59 (2.6)	51 (3.7)	29 (2.7)	33 (3.1)	20 (2.4)
Body mass index, n (%)	27.9 (5.6)	27.6 (5.7)	27.2 (6.3)	26.8 (6.3)	28.7 (4.7)	28.5 (4.8)
<18.5 kg/m^2^	26 (0.9)	25 (1.1)	22 (1.6)	19 (1.8)	3 (0.3)	4 (0.5)
18.5–24	902 (31.2)	798 (34.4)	553 (40.3)	474 (44.1)	203 (19.3)	178 (21.3)
25–29	1226 (39.0)	878 (37.9)	441 (32.1)	325 (30.3)	513 (48.7)	409 (48.9)
30–34	531 (18.4)	381 (16.4)	209 (15.2)	144 (13.4)	228 (21.7)	165 (19.7)
35–39	196 (6.8)	153 (6.6)	84 (6.1)	65 (6.1)	80 (7.6)	61 (7.3)
≥40	102 (3.5)	79 (3.4)	62 (4.5)	45 (4.2)	25 (3.4)	20 (2.4)
Missing	5 (0.2)	3 (0.1)	3 (0.2)	2 (0.2)	1 (0.1)	0 (0.0)
Waist circumference, in	38.2 (6.0)	37.4 (6.0)	36.8 (6.4)	35.7 (6.3)	40.0 (4.9)	39.4 (5.0)
Physical activity, n (%)						
<32.7	650 (22.5)	541 (23.4)	297 (21.6)	239 (22.3)	246 (23.4)	194 (23.2)
32.7–36.1	678 (23.5)	537 (23.2)	338 (24.6)	269 (25.1)	227 (21.6)	177 (21.2)
36.2–42.0	697 (24.1)	566 (24.4)	338 (24.6)	261 (24.3)	245 (23.3)	205 (24.5)
≥42.1	750 (26.0)	613 (26.5)	338 (24.6)	273 (25.4)	300 (28.5)	242 (28.9)
Missing	113 (3.9)	60 (2.6)	63 (4.6)	32 (3.0)	35 (3.3)	19 (2.3)
CES-D ≥16, n (%)	473 (16.4)	451 (19.5)	248 (18.1)	220 (20.5)	155 (14.7)	155 (18.5)
Not employed outside home, n (%)	697 (24.1)	304 (13.1)	427 (31.1)	204 (19.0)	169 (16.1)	50 (6.0)
CVD risk, %	9.7 (10.6)	24.2 (17.3)	6.6 (7.6)	19.2 (15.0)	13.7 (12.7)	30.5 (17.8)

Abbreviations: CES-D, Center for Epidemiologic Studies of Depression Scale; CVD, cardiovascular disease; FRS, Framingham risk score; SD, standard deviation.

All values are presented as mean (SD) unless otherwise noted

Little of the cross-sectional variation in the age- and sex-independent 10-year FRS was attributable to the Census block group-level (Table 2). The ICC for the 10-year FRS among all participants was 1.73% (95% CI: 0.62, 4.72%). ICC estimates were too imprecise to distinguish changes upon addition of covariates. The ICC was reduced to 0.91% (95% CI: 0.17, 4.84%). 1.15 (95% CI: 0.29, 4.49%), and 0.69 (95% CI: 0.07, 6.77%) when education, green space, and all individual- and neighborhood-level variables were added to the model, respectively. Among women, the neighborhood-level ICC was 2.70% (95% CI: 0.93, 7.56), while it was negligible among men.

**Table 2:** Variance of 10-year and 30-year CVD risk scores explained by neighborhood membership, offspring cohort examination 7 (1998–2001)/generation 3 examination 1 (2002–2005).

	10-year FRS			30-year FRS		
	N_P_	N_N_	ICC (%)	N_P_	N_N_	ICC (%)
All participants	2888	187		2317	164	
Empty model			1.73 (0.62, 4.72)			3.31 (1.66, 6.47)
+ Education			0.91 (0.17, 4.84)			2.28 (0.95, 5.37)
+ BMI			1.41 (0.39, 5.02)			2.96 (1.38, 6.25)
+ Waist circumference			1.52 (0.45, 5.02)			2.83 91.29, 6.13)
+ Physical activity			1.69 (0.60, 4.67)			3.28 (1.65, 6.44)
+ CES-D≥16			1.67 (0.58, 4.71)			3.09 (1.50, 6.25)
+ Green space			1.15 (0.29, 4.49)			2.41 (1.01, 5.66)
+ # of intersections			1.73 (0.63, 4.67)			3.28 (1.64, 6.42)
+ # of food stores			1.71 (0.60, 4.73)			3.16 (1.55, 6.34)
+ All variables			0.69 (0.07, 6.77)			1.20 (0.26, 5.26)
Women	1374	117		1074	101	
Empty model			2.70 (0.93, 7.56)			6.47 (3.22, 12.58)
+ Education			1.83 (0.44, 7.26)			5.09 (2.27, 11.04)
+ BMI			1.27 (0.17, 8.93)			4.12 (1.52, 10.70)
+ Waist circumference			1.62 (0.33, 7.67)			3.20 (0.98, 9.93)
+ Physical activity			2.66 (0.91, 7.50)			6.44 (3.20, 12.55)
+ CES-D≥16			2.83 (1.00, 7.76)			5.94 (2.83, 12.01)
+ Green space			1.52 (0.28, 7.87)			4.75 (1.92, 11.25)
+ # of intersections			2.64 (0.90, 7.50)			6.37 (3.16, 12.45)
+ # of food stores			2.23 (0.64, 7.49)			5.51 (2.49, 11.73)
+ All variables			0.91 (0.06, 12.31)			1.08 (0.06, 16.37)
Men	1053	94		837	84	
Empty model			0.23 (0.00, 99.47)			0.74 (0.01, 33.31)
+ Education			0.03 (0.00, 100.00)			0.33 (0.00, 96.01)
+ BMI			0.00 ()			0.14 (0.00, 100.00)
+ Waist circumference			0.20 (0.00, 99.97)			0.18 (0.00, 100.00)
+ Physical activity			0.22 (0.00, 99.77)			0.83 (0.02, 28.57)
+ CES-D≥16			0.28 (0.00, 97.50)			0.71 (0.01, 39.32)
+ Green space			0.12 (0.00, 100.00)			0.00 ()
+ # of intersections			0.10 (0.00, 100.00)			0.88 (0.02, 25.28)
+ # of food stores			0.00 ()			0.38 (0.00, 89.67)
+ All variables			0.00 ()			0.00 ()

Abbreviations: ICC, intraclass correlation (% of variance explained by neighborhood residence); FRS, Framingham Risk Score; N_N_, number of neighborhoods; N_P_, number of participants.

All models contain only the variables listed.

The proportion of the variance in the 30-year FRS was explained at the neighborhood level appeared to be greater than that for the 10-year FRS (Table 2). The ICC for the 30-year FRS was 3.31% (95% CI: 1.66, 6.47%). As was the case with 10-year FRS, addition of education and green space appeared to result in the largest reductions in ICC, but confidence intervals were wide. In sex-stratified analyses, ICCs were 6.47% (95% CI: 3.22, 12.58%) and 0.74% (95% CI: 0.01, 33.31%), respectively. Among women, addition of waist circumference appeared to result in the greatest reduction in neighborhood-level variance (ICC: 3.20%, 95% CI: 0.98, 9.93%).

## Discussion

In our cross-sectional analysis of the contribution of neighborhood of residence to the variance in 10-year and 30-year FRS, we observed little variation at the neighborhood level. When we conducted analyses stratified by sex, neighborhoods explained approximately 2.7% and 6.5% of the 10- and 30-year FRS among women, but essentially no variance among men.

Although imprecisely estimated, our results generally agree with prior studies which reported modest (approximately 2–6%) amounts of neighborhood-level variation in CVD risk. Chaix et al. observed unadjusted ICCs of 12% and 3% for incidence of ischemic heart disease (IHD) among Swedes aged 50–64 and 65–79, respectively, finding that more urban, deprived areas had higher IHD incidence [6]. A study of coronary heart disease (CHD) reported sex-stratified adjusted ICCs of 2.1% for women and 0.9% and for men [3]. Another study of the associations of neighborhood resources and the incidence and mortality of CHD and stroke reported unadjusted ICCs ranging from 0.4 to 1.0%, depending on the outcome [4]. After accounting for individual-level factors, Hart et al. observed significant district-level variances of 2.6% and 2.4% for SBP and 0.5% and 1.0% for serum cholesterol among men and women, respectively, in Scotland [5]. For comparison, the variance in in CVD-associated phenotypes explained by genetic risk scores range from around 3% for hypertension to 6% for coronary artery calcium [18].

Our results indicated that the cross-sectional neighborhood-level ICC was larger for women than for men. This is consistent with results from two prior studies which presented results stratified by sex [3, 5]. Among studies that have investigated associations of neighborhood built environment and CVD risk factors, one observed a greater proportion of the variance of obesity explained at the neighborhood level among men compared with women [19]. In addition, studies of associations of changes in the number of physical activity facilities and supermarkets with change in BMI [20], and of walkability with change in waist circumference [21], reported associations only among men. A study of neighborhood built- and social environments observed an association between the neighborhood social environment and incident diabetes among women, but not men [22]. Investigations of the associations of health-promoting resources with obesity [23], fast food availability with BMI [24], and healthy food availability and neighborhood walkability with diabetes [25] reported no differences in associations by sex.

We consistently observed higher neighborhood-level proportional variance explained for the 30-year FRS compared with the 10-year FRS. This is consistent with the study by Chaix et al investigating ischemic heart disease, where the ICC for IHD incidence was 4-fold higher among 50–64 year old participants compared with those aged 65–79 [6]. Studies of neighborhoods and mortality have also observed diminished associations among older adults compared with younger individuals [26, 27]. Explanations of these phenomena have included survivor bias resulting in observing only relatively healthier older adults [6, 26, 27], the greater proportion of total residential history represented by a single observation of residential neighborhood for younger people [26], and the higher baseline risk among older adults [6, 26, 27].

The ways in which neighborhoods may differentially affect men versus women are unclear. A cross-sectional study reported an association between the neighborhood food environment and fruit and vegetable intake only among those spending moderate and long periods of time at home, with women spending over an hour more at home than men, on average [28]. Of note, women in our study were less likely to work outside the home. The specific mechanisms linking neighborhood to CVD risk are likely to be important as well. For example, mechanisms mediated by weight may have a lower impact on premenopausal women, who tend to have a more metabolically favorable distribution of adipose tissue [29]. Alternatively, mechanisms which act through stress may have a greater influence in women, among whom stress responses are, on average, reported to be somewhat stronger [30].

In the main analysis, the point estimate for the ICC was lowest when percentage of green space was added to the model. Among women, adding waist circumference and density of food stores to the model appeared to reduce the ICC of cross-sectional FRS by a similar amount. Among men, the variable which appeared to have the greatest impact on the ICC for change in FRS was the number of food stores, while there was little change in the ICC upon inclusion of the other variables investigated. All these estimates were too imprecise to make definitive statements about the relative impact of the inclusion of specific covariates in the models, so these observations must be interpreted cautiously.

If neighborhoods influence outcomes such as diet, physical activity, obesity, and diabetes, why might the variance explained be so low in our report? Several possible explanations exist. In general, studies of the built environment are challenged by the problem of defining neighborhoods. Misclassification of exposure is a large problem, as the relevant geographic scales are unknown, may be highly variable among individuals, and may differ according to the exposure of interest [1]. Neighborhood exposures at different ages may also differ in their relevance to adult health [31]. Administrative boundaries, such as those used in this analysis, have been both widely used and criticized as neighborhood proxies [1, 2, 31]. In addition, variance explained is not equivalent to causation. If there is too little variation in relevant neighborhood-level factors (certainly plausible in our study), they cannot possibly cause variation in the outcome [2]. Finally, if exposures only affect a disease via their influence on intermediates that occur early in a disease process (e.g., diet or physical activity), even strong effects on those intermediates may translate into small effects on the outcomes of interest [32].

Advantages of the current study include the collection of study-measured data and the use of a well-validated risk score to measure CVD risk. One limitation is the use of administrative boundaries as proxies for neighborhoods. In addition, the exclusion of participants who lived in otherwise eligible neighborhoods which lacked a sufficient number of participants may have influenced the results by reducing the overall heterogeneity of the included neighborhoods or resident participants. This would likely be an even greater problem in the sex-stratified analysis. We lacked sufficient power to distinguish the impacts of the inclusion of individual- and neighborhood-level results on the proportion of FRS explained by neighborhood-level variance. Our results are cross-sectional, and may not reflect the importance of neighborhood over the life course. Additionally, results from this overwhelmingly white population who have remained in or moved back to Massachusetts may lack external validity. There may be considerable geospatial variation in the contribution of the built environment to CVD risk across the continental United States.

Although our results must be interpreted with caution (given the limitations noted above), our findings suggest that, in this relatively homogenous, primarily suburban white population, neighborhood contributes relatively little to CVD risk as estimated by the 10-year and 30-year FRS. Our data suggest that a hypothetical intervention to improve the neighborhood context to the best level available within the study may have minimal impact on CVD risk in this population (although other benefits may well accrue).
